# The Impact of HLA-C Matching on Donor Identification Rates in a European-Caucasian Population

**DOI:** 10.3389/fimmu.2014.00501

**Published:** 2014-10-15

**Authors:** Hans-Peter Eberhard, Carlheinz R. Müller

**Affiliations:** ^1^Zentrales Knochenmarkspender-Register Deutschland (ZKRD), Ulm, Germany

**Keywords:** HLA-C, haplotype frequency estimation, donor registry, hematopoietic stem cell transplantation, donor-patient matching, donor identification rate

## Abstract

The degree of HLA concordance with the patient has long been known to be the major donor-related prediction factor for the success of hematopoietic stem cell transplantations and, with the progress of HLA typing technology, selection criteria became more stringent with regard to the recommended loci and resolution. A late refinement was HLA-C matching, which gained broader acceptance only after the turn of the millennium. The enormous HLA polymorphism has always necessitated registries with a large number of donors in order to be able to provide well-matched donors to a substantial fraction of patients. Using a biostatistical approach, we investigated the impact of adding HLA-C at low or high resolution as a supplementary matching criterion on some key parameters in donor provision for a European-Caucasian population. Starting point is donor selection based on allele level matching for HLA-A, -B, -DRB1, and, optionally, HLA-DQB1. Without typing for HLA-C, 68% of the donors selected based on matching for HLA-A, -B, -DRB1, and -DQB1 at high resolution will also match for HLA-C, 29% will have a single and only 3% will have two HLA-C alleles different from the patient. In order to provide the same fraction of patients with a fully matched donor, a registry would have to be about twice the size if HLA-C is considered in addition to the four other loci, with the exact factor increasing with the registry’s size. If the provision of donors with up to a single allele mismatch is considered, this factor doubles due to the strong linkage between HLA-B and -C. These figures only change slightly when HLA-DQB1 is completely ignored or HLA-C matching is only considered at low resolution. Our results contribute to quantifying the medical and economic impact of the progress in donor selection algorithms.

## Introduction

Allogeneic hematopoietic stem cell transplantation (HSCT) is a well-established therapy with curative potential for malignant diseases of the blood and numerous otherwise fatal non-malignant disorders (1, 2). The degree of HLA matching with the patient has long been known to be the major donor-related factor predicting the success of a HSCT (3–5). With the progress of HLA typing technology during the last decades, the number of loci and the typing resolution used in donor selection have continuously increased. Typing and matching for HLA-A, -B, and -DR(B1) was a long-term practice in the 1980s and 1990s when serological and cellular testing were gradually replaced by molecular technology, first for class II and later for class I, boosting the reproducibility and resolution of typing results (6, 7). While the attention paid to HLA-DQB1 has varied, in spite of earlier indications of its relevance (8) HLA-C was largely neglected until after the turn of the millennium (9, 10). In earlier days, the low quality of serological HLA-C testing, the strong HLA-B, -C linkage, and the habit of experts to misuse this linkage in the interpretation of inconclusive laboratory data for HLA-C hampered establishing HLA-C as transplantation antigen. After the analyses of Flomenberg, Lee, and Woolfrey (11–13) during the last decade, HLA-C is now routinely considered in donor selection (14–16), but discussions are still ongoing as to if doing so at low resolution would be sufficient (13, 17).

Haplotype frequency estimation (HFE) from phenotypic population data is well-established (18–20) and the challenges posed by the size of registry-derived datasets and their inherent ambiguities and heterogeneities have intensively been studied (20, 21). In return, these haplotype frequencies (HF) can be used to study certain population properties, in particular, the dependence of the supply of donors on the size of a registry of volunteers from the population (19, 22–25).

In this study, we will investigate the impact of HLA-C matching on the donor procurement rates in a European-Caucasian population as well as the growth required for a registry to keep up its procurement rate when confronted with more stringent HLA-C matching criteria. Due to the heterogeneity of common practice with regard to HLA-DQB1 (15, 26), we will present results for matching procedures including and disregarding HLA-DQB1.

## Materials and Methods

We have estimated five-locus HLA-A, -B, -C, -DRB1, -DQB1 HF at high resolution (i.e., using the first two fields of the allele names) from a snapshot of the German donor database at the ZKRD (Zentrales Knochenmarkspender-Register Deutschland, i.e., the German National Stem Cell Donor Registry) from May 2014, including 2,001,575 individuals with HLA designations at molecular level for HLA-A, -B, -C, -DRB1, and -DQB1. All HLA assignments used are based on the IMGT/HLA Database Release 3.16.0, 2014-04-14 (27) and the World Marrow Donor Association guidelines for the use of the HLA nomenclature (28, 29).

The HFE was performed using our well-established (19, 21) and validated (30) HFE program, which was further tuned to handle the increased ambiguity and size of this sample. This allowed for the inclusion of all donors with molecular HLA typing data at any resolution, leading to a sample size of over 2 million individuals. Allelic ambiguities were dealt with as described in (21). From this dataset, the frequencies of 89,474 five-locus haplotype at high resolution have been estimated. Since variations outside the antigen recognition site (ARS, i.e., exon 2 and 3 for HLA class I and exon 2 for HLA class II) are typically not taken into account for donor selection (31–33), we have collapsed the frequencies of ARS identical haplotypes yielding a final set of 83,446 ARS-level HF.

The probability of a random patient finding at least one suitably matched donor within a given population of donors can be calculated as

$$C\left(n\right)=\sum_{i=1}^{P}p_{i}\times \left(1-{\left(1-d_{i}\right)}^{n}\right)$$

where *P* is the number of defined phenotypes, *p_i_* is the frequency of the phenotype in the patient population, *d_i_* is the cumulative frequency of the suitably matched donors for this phenotype in the donor population, and *n* is the number of donors in the registry (24). In this calculation, the frequencies *p_i_* and *d_i_* are easily derived from the previously obtained HF and the result *C(n)* indicates the extent to which the patient population is “covered” by a registry of *n* donors. We will use the term “coverage” to refer to *C(n)*, depending on the registry size *n* and on the resolution of the haplotypes of the underlying HF vector. The estimation of HF as well as the calculation of phenotype frequencies from HF assumes that the examined population is reasonably close to Hardy–Weinberg equilibrium (HWE) and we know from previous studies (19, 21) that the German donor population does not substantially deviate from the HWE assumption.

In order to analyze various matching scenarios, from the full set of 83,446 HF, we derived several marginal HF vectors by mapping HLA-C alleles to low resolution (i.e., using only the first field of the allele name) or dropping HLA-C and/or HLA-DQB1 completely from the calculations performed. Moreover, we allowed one or two mismatches in the matching criteria used to select the donors contributing to the frequencies reflected in *d_i_* above.

All calculations were performed on an IBM x3550 server with 192 GB RAM and 24 CPU kernels running at 3.47 GHz under Ubuntu Linux 14.04. All time-critical parts of the HFE and the coverage calculations were written in C, whereas simpler tasks, like data pre- and post-processing, were implemented in Perl. Statistical analysis was performed with supportive Perl programs, standard spreadsheet software and the open source statistical package “R” (34).

## Results

**Figure 1:**
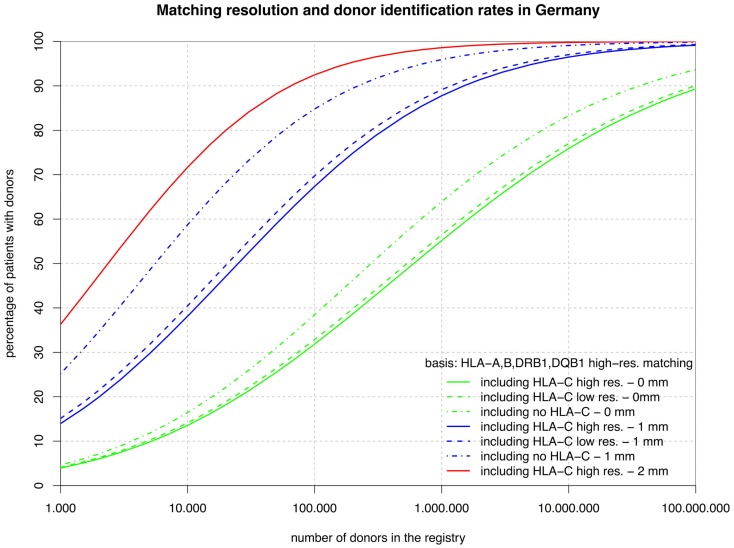
**The curves show the percentage of patients capable of finding a matched donor depending on the size of the registry (horizontal axis in logarithmic scale) according to various typing resolutions (HLA-C matching considered at high or low resolution or not at all; continuous, dashed, dashed-and-dotted) and the maximum number of mismatches allowed (0, 1, or 2; green, blue, or red)**. All matching includes HLA-A, -B, -DRB1, and -DQB1.

**Figure 2:**
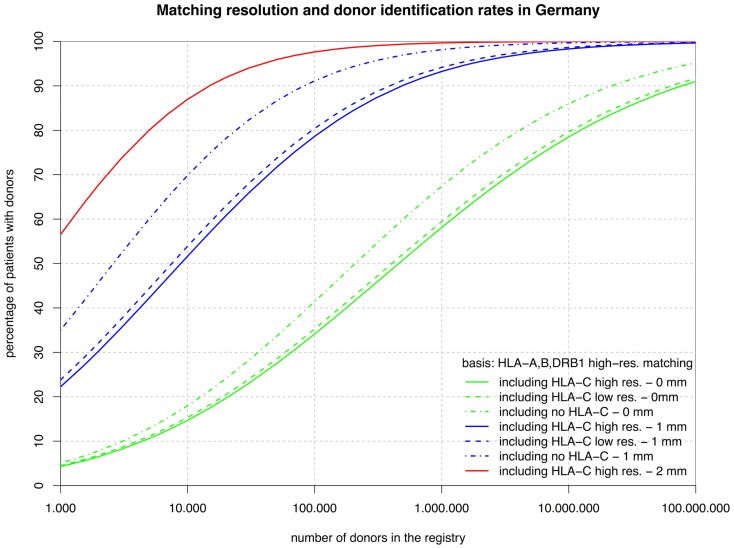
**The curves show the percentage of patients capable of finding a matched donor depending on the size of the registry (horizontal axis in logarithmic scale) according to various typing resolutions (HLA-C matching considered at high or low resolution or not at all; continuous, dashed, dashed-and-dotted) and the maximum number of mismatches allowed (0, 1, or 2; green, blue, or red)**. Here, all matching includes HLA-A, -B, and -DRB1, but not HLA-DQB1.

From the set of five-locus high-resolution HF, we have derived five related sets of marginal frequencies. The six frequency vectors are based on HLA-A, -B, -DRB1 at high resolution with C missing or added at low or high resolution and, independently, HLA-DQB1 added or omitted. For all six variants, we report the coverage based on full matches (green curves in Figures 1 and 2; lines numbered 1–3 in Table 1) and single mismatches (blue curves; lines numbered 4–6 in Table 1); however, we show the two mismatch coverage only for the two variants including HLA-C at high resolution (see also left part of Table 1).

**Table 1:** **This table shows the attributes of the seven matching variants underlying the curves in Figures 1 and 2 and some of their key coordinates, i.e., the number of donors required to reach a coverage of 25, 50, 75, and 90% (numbers below 1,000 and over 100,000,000 have been blanked due to practical irrelevance and are also not reflected in the figures)**.

Curve	Matching criteria		Maximum no. of mismatches	Thousand donors required to cover patient fraction of			
	HLA-A, -B, -DRB1	HLA-C	Allowed	25%	50%	75%	90%
Figure 1						
1	Plus DQB1	High res.	0	47	602	8,879	
2	Plus DQB1	Low res.	0	42	533	7,675	98,546
3	Plus DQB1	No	0	27	277	3,251	35,763
4	Plus DQB1	High res.	1	3	25	202	1,492
5	Plus DQB1	Low res.	1	3	20	163	1,171
6	Plus DQB1	No	1	1	6	35	218
7	Plus DQB1	High res.	2		2	13	64
Figure 2						
1	No DQB1	High res.	0	38	458	6,292	77,603
2	No DQB1	Low res.	0	34	404	5,414	64,001
3	No DQB1	No	0	22	208	2,225	21,797
4	No DQB1	High res.	1	1	9	69	485
5	No DQB1	Low res.	1	1	7	56	385
6	No DQB1	No	1		3	15	82
7	No DQB1	High res.	2		1	3	14

The sets of lines with identical color in Figures 1 and 2 illustrate the influence of adding HLA-C at low and high resolution as a matching criterion to high-resolution matching for HLA-A, -B, -DRB1, -DQB1, and HLA-A, -B, -DRB1. These graphs allow for a number of very interesting observations:

- The small difference between the dashed and the continuous lines of the same color demonstrate that the HLA-C polymorphism is already largely revealed at low resolution among individuals allele-matched for the other three or four loci.
- The large distance between the dashed-and-dotted green lines and the other two green lines shows substantial further discrimination added by HLA-C at any resolution to three- or four-locus allele matching.
- Likewise, the small difference between the corresponding green lines in the two figures reflects the strong linkage between HLA-DRB1 and -DQB1, also explaining the long ongoing debate about the relevance of HLA-DQB1 as a criterion in HSCT.
- In contrast, the substantial difference between the corresponding blue curves (one mismatch) in the two figures is quite a surprise. The reason for this is primarily, again, the strong HLA-DRB1-DQB1 linkage that shifts a substantial part of the donors mismatched for HLA-DRB1 from the blue curves of Figure 2 (one mismatch without considering HLA-DQB1) into the higher red curve of Figure 1 (two mismatches) and, as a consequence, brings down the blue curves.
- The red curves show that a registry of 1 million donors would provide over 98% of the patients with an 8/10 donor and over 99% with a 6/8 donor (i.e., two high-resolution mismatches, considering or ignoring HLA-DQB1). The distance from the dashed-and-dotted blue line reflects donors fully matched for HLA-C, but with two mismatches at other loci.

Now, we can investigate the quantitative details of these curves and first consider the vertical distance between the green curves. Adding HLA-C matching at a registry size of 10,000 donors only reduces the coverage compared with fully matched donors by 3% points, but this gap increases to almost 10% points beyond 1 million donors.

Conversely, considering the horizontal distance between the green curves, maintaining the same coverage with the higher matching requirements demands increasing the registry size. For fully matched donors, the increment required is a factor between two and three, whereas for single-mismatched donors this factor ranges from three to around seven. Since this cannot easily be measured in the two figures due to the logarithmic scale of the *x*-axis, we have added the four columns on the right of Table 1, revealing these ratios.

As a by-product of the coverage calculations, we observed that 68% of the donors selected using high-resolution matching for HLA-A, -B, -DRB1, -DQB1 are also a full match for HLA-C, 29% will have a single mismatch and only 3% will have a double mismatch and even without including HLA-DQB1 upfront, the corresponding numbers are virtually identical (67, 30, and 3%).

## Discussion

The important role of HLA-C matching for a successful HSCT is well accepted today (5, 12, 13); however, the impact of a HLA-C difference for the recipient may depend on stem cell source and the type of mismatch present. The fact that over two-thirds of the donors selected on the basis of HLA-A, -B, -DRB1 high-resolution matching while omitting HLA-C are indeed HLA-C identical by chance has contributed to the success of HSCTs preceding HLA-C matching.

However, there is an important complementary aspect to this. Figure 1 shows that, at a realistic registry size of 1 million donors, 64% of the patients have an HLA-A, -B, -DRB1, -DQB1 identical donor and 55% of the patients even have a donor that is also identical for HLA-C. Without typing for HLA-C only 44% of the patients would get a five-locus identical donor by chance. Therefore, more than half, i.e. (55–44%)/(64–44%), of the remaining mismatch situations could be avoided without increasing the registry size merely by including HLA-C as a primary selection criterion.

Maintaining the same rate of fully matched donors including HLA-C requires increasing the size of a donor registry by a factor of two to three. This may be achieved in European countries in the near future if the forthcoming revolution in HLA typing by next generation sequencing will hold its pricing promises. However, if single mismatch transplants are considered, the step from ≥7/8 to ≥9/10 matching is substantially higher since many HLA-B mismatches are typically accompanied by the corresponding HLA-C mismatch so that the size of the donor file would have to grow by a factor of three to seven to maintain the previous procurement rate.

Over 98% of the patients would have a ≥8/10 identical donor in a registry of 1 million donors so that accepting a second mismatch would almost completely resolve this problem. Unfortunately, such donors are still associated with substantially inferior outcome (5, 12, 13).

Basically, the results we have obtained underline the feasibility of including HLA-C as a primary matching criterion in donor selection for a European-Caucasian population today and, at the same time, justify and stimulate the further growth of all donor registries.

Furthermore, we were able to quantify the impact of the resolution of HLA-C typing on the identification rate of matched donors. Theoretically, in the context of matching for four other loci including HLA-B, low resolution HLA-C already reflects most of the additional polymorphism. As a consequence, it will remain difficult to decide if HLA-C matching at low resolution is sufficient or not. In practice, however, HLA-C typing should always be performed upfront for donors at the highest affordable resolution to expedite the search process and to avoid unnecessary HLA-C mismatching (17). With regard to the permissible HLA-C mismatch HLA-C*03:03 vs. 03:04 discussed in (35), a variant of the calculations reported here shows that in more than half of the cases where a low resolution HLA-C mismatch can be avoided but a high-resolution HLA-C mismatch must be accepted, this mismatch can be chosen to be HLA-C*03:03 vs. 03:04.

It is quite likely that these simulations based on a German population, with little recent admixture, will basically hold true for many similar populations of European-Caucasian background. However, it would be extremely interesting how these HLA-C-related observations translate into fundamentally different racial/ethnical groups, like those studied in (36). Unfortunately, similar investigations at the global level are still hampered by the lack of accessible multi-locus high-resolution HLA data for most populations as well as their corresponding donor availability rates. So a more realistic modeling of global donor procurement rates remains a challenge for the future.

## Conflict of Interest Statement

The authors declare that the research was conducted in the absence of any commercial or financial relationships that could be construed as a potential conflict of interest.
